# Pediatric and young adult ovarian masses: clinical approach, diagnostic evaluation, and management

**DOI:** 10.3389/fped.2025.1639582

**Published:** 2025-09-11

**Authors:** Zeynep Bayramoglu, Buket Timur, Deniz Kızmazoglu, Hikmet Tunc Timur, Oktay Ulusoy, Safiye Aktas, Nur Olgun, Sefa Kurt

**Affiliations:** ^1^ Department of Pathology, Dokuz Eylül University Faculty of Medicine Hospital, Izmir, Türkiye; ^2^ Department of Molecular Pathology, Institute of Oncology, Dokuz Eylül University, Izmir, Türkiye; ^3^ Division of Pediatric Oncology, Department of Clinical Oncology, Institute of Oncology, Dokuz Eylül University, Izmir, Türkiye; ^4^ Department of Obstetrics and Gynecology, Division of Gynecological Oncology, Dokuz Eylül University Faculty of Medicine Hospital, Izmir, Türkiye; ^5^ Department of Pediatric Surgery, Dokuz Eylül University Faculty of Medicine Hospital, Izmir, Türkiye

**Keywords:** ovary, pediactric, young adolescence, mass, maliganant tumor

## Abstract

**Objective:**

To evaluate the clinical, pathological, and surgical characteristics of ovarian masses in pediatric and young adult patients, with emphasis on malignancy risk, surgical approach, recurrence, and fertility outcomes.

**Materials and methods:**

This retrospective cohort study included 1,128 female patients under the age of 30 who underwent surgery for ovarian masses between 2003 and 2024. Clinical presentation, imaging, tumor markers, surgical procedures, histopathology, and recurrence were analyzed.

**Results:**

The mean age of patients was 13.7 ± 4.02 years. Right-sided masses were more common (69.6%), and 79.9% of surgeries were open. Benign tumors were predominant (most commonly mature cystic teratomas), while dysgerminomas were the most frequent malignant neoplasms. Tumor size was significantly larger in malignant cases (*p* < 0.005). AFP and *β*-hCG demonstrated high specificity (88% and 90%, respectively) in predicting malignancy. Fertility-sparing surgery was performed in a large proportion of cases. Recurrence was observed in 31% of borderline tumors, 33% of grade 2–3 immature teratomas, 5% of grade 1 immature teratomas, and 12% of malignant germ cell tumors. Laparoscopic procedures, performed in 20% of patients, were associated with better ovarian preservation. Due to the retrospective design, long-term fertility outcomes were not systematically available.

**Conclusion:**

Ovarian masses in pediatric and young adult patients are mostly benign, but a notable risk of malignancy remains, especially in older adolescents and young adults. Tumor markers and imaging aid in preoperative risk stratification. Fertility-sparing surgery is feasible and should be prioritized. However, recurrence rates vary by histology, highlighting the need for structured long-term follow-up in this population.

## Introduction

Although rare, ovarian masses in the pediatric and adolescent population present distinct clinical and pathological features compared to adults (1, 2). These lesions range from simple functional cysts to benign neoplasms and malignant tumors. Malignant ovarian tumors account for only about 1% of all pediatric cancers but are of critical importance due to the need for accurate diagnosis and effective management (3). Therefore, a comprehensive understanding of the clinical behavior of these lesions and the development of age-appropriate treatment strategies is essential.

The differential diagnosis of ovarian masses poses a significant clinical challenge due to the difficulty in distinguishing between benign and malignant lesions. A reliable diagnostic approach is crucial to assess malignancy risk and guide treatment planning. Ultrasonography (US) is typically the first-line imaging modality but has limitations in differentiating benign from malignant lesions (4). In cases with suspicion of malignancy, advanced imaging techniques such as computed tomography (CT) and magnetic resonance imaging (MRI) provide valuable diagnostic information and assist in surgical planning (5). Additionally, serum tumor markers (e.g., AFP, CA125, CA19-9, CEA, and β-hCG) support malignancy assessment (6–8), although they are not definitive on their own. Notably, elevated AFP levels are highly specific for malignant germ cell tumors (9). Tumor size is also a significant risk factor, with larger masses being more frequently associated with malignancy (10).

The primary objective in managing ovarian masses in pediatric and adolescent patients is to achieve malignancy control while preserving gonadal function and, consequently, fertility potential (11). Surgery remains the cornerstone of treatment (12). In benign cases, ovary-sparing techniques such as cystectomy are commonly preferred. Laparoscopic surgery has been shown to offer higher ovarian preservation rates compared to open surgery (13). However, oophorectomy may be necessary in the case of large masses or high suspicion of malignancy. Acute complications such as ovarian torsion require urgent surgical intervention, and adjuvant chemotherapy is generally needed for malignant tumors (14).

Long-term health risks and potential genetic predispositions must also be considered in the management of ovarian masses (15). The risk of secondary primary malignancies following treatment of the initial malignancy is a significant concern in this age group (16). Certain ovarian tumors are associated with hereditary cancer predisposition syndromes (17). Thus, treatment planning for young patients should not only address the current tumor but also include long-term health monitoring and genetic evaluation. In line with modern oncologic principles, fertility-preserving strategies (oncofertility) should be prioritized (18).

In this study, we aim to provide a comprehensive analysis of the clinical presentation, diagnostic evaluation, and surgical management of ovarian masses in children and adolescents, thereby contributing to the optimization of management strategies specific to this population.

## Materials and methods

A retrospective review was conducted on 1,128 female patients under the age of 30 who underwent surgery for ovarian masses at the Departments of Pediatric Surgery and Obstetrics and Gynecology between 2003 and 2024.

Patient data were collected from medical records, including demographic characteristics, clinical symptoms and findings, laboratory test results, details of the surgical procedure, the presence of ovarian torsion, and histopathological diagnoses. Based on pathology reports, final diagnoses were initially categorized into non-neoplastic and neoplastic groups, with the neoplastic group further subclassified into benign and malignant tumors.

### Statistical analysis

Descriptive statistics (mean, standard deviation, median, percentages) were used to summarize the data. Differences between groups were analyzed using appropriate statistical tests, including chi-square tests, non-parametric Mann–Whitney *U* tests, and multivariate logistic regression analysis.

The diagnostic performance of serum tumor markers was assessed by calculating their sensitivity, specificity, positive predictive value (PPV), and negative predictive value (NPV) using 2 × 2 contingency tables. Statistical significance was defined as *p* < 0.05. Statistical analyses were performed using the SPSS (Statistical Package for the Social Sciences) version 22.0 software package.

## Results

The study included a total of 1,128 patients, with a mean age of 13.7 ± 4.02 years. Among these, 786 (69.6%) had lesions localized to the right ovary, 230 (20.3%) to the left ovary, and 112 (9.9%) had bilateral involvement. A total of 902 patients (79.9%) underwent open surgery, while 226 (20.0%) underwent laparoscopic procedures. Postmenarchal patients accounted for 896 cases (79.4%), and 232 patients (20.5%) were premenarchal. The clinicopathological characteristics of the patients are summarized in Table 1.

**Table 1:** Descriptive statistics.

Lesion location		
Lesion location	Number	Percentage (%)
Right ovary	786	69.68
Left ovary	230	20.39
Bilateral	112	9.93
Surgical procedure		
Surgical procedure	Number	Percentage (%)
Open surgery	902	79.96
Laparoscopic surgery	226	20.04
Menarche status		
Menarche status	Number	Percentage (%)
Post-menarche	896	79.43
Pre-menarche	232	20.57
Pathological diagnosis		
Pathological diagnosis	Number	Percentage (%)
Non-neoplastic	760	67.38
Neoplastic	368	32.62

The mean age was 15.68 ± 4.67 years in the benign group and 21.45 ± 3.31 years in the malignant group. No statistically significant difference was found between the two groups in terms of age at presentation. However, a significant difference was noted in tumor size, with malignant neoplasms being significantly larger than benign ones (*p* < 0.005; Table 2).

**Table 2:** Comparison of characteristics of benign and malignant neoplasms.

Characteristics	Benign neoplasm (*n*:)	Malignant neoplasm (*n*:)	*p*-value
Age at presentation	15.6 ± 8.4	21.4 ± 5.3	0.124
Tumor size (cm)	6.3 ± 5.7	18.4 ± 8.5	0.001

Various surgical procedures were employed: cyst excision was performed in 733 patients (64.9%), salpingo-oophorectomy in 230 patients (20.3%), and detorsion with cyst excision in 112 patients (9.9%). Among the 112 patients who underwent surgery for ovarian torsion, 47 showed decreased or absent ovarian vascularization on preoperative ultrasonography. Detorsion was performed in 57 of the 112 torsion cases. Ovarian torsion was also present in 10 patients diagnosed with malignancy.

**Figure 1:**
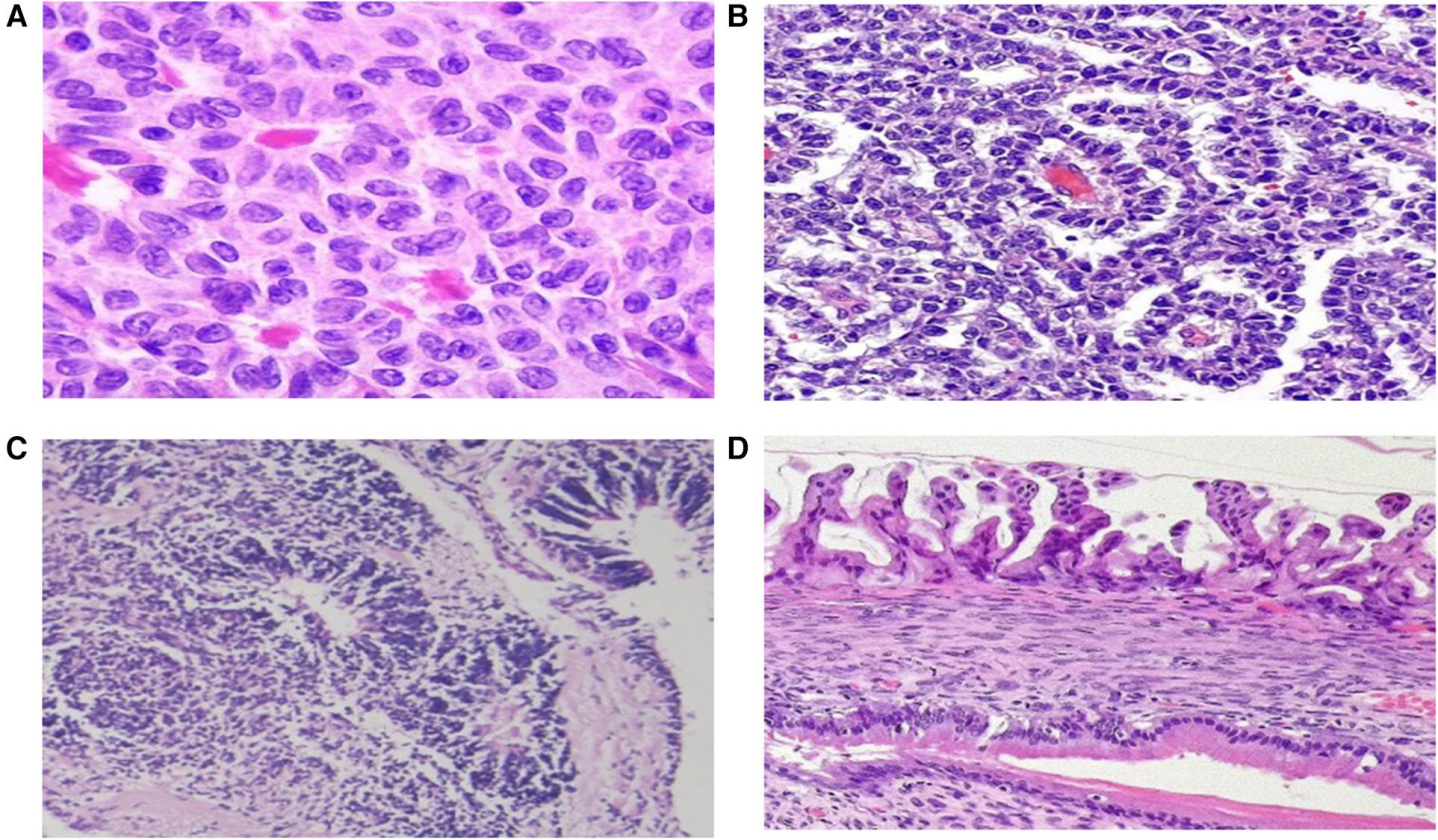
Microscopic images of ovarian tumors. **(A)** Call-Exner bodies in an adult-type granulosa cell tumor, **(B)** Schiller-Duval bodies in a yolk sac tumor, **(C)** immature teratoma, **(D)** Borderline mucinous ovarian tumor. (Hematoxylin and eosin stain).

**Figure 2:**
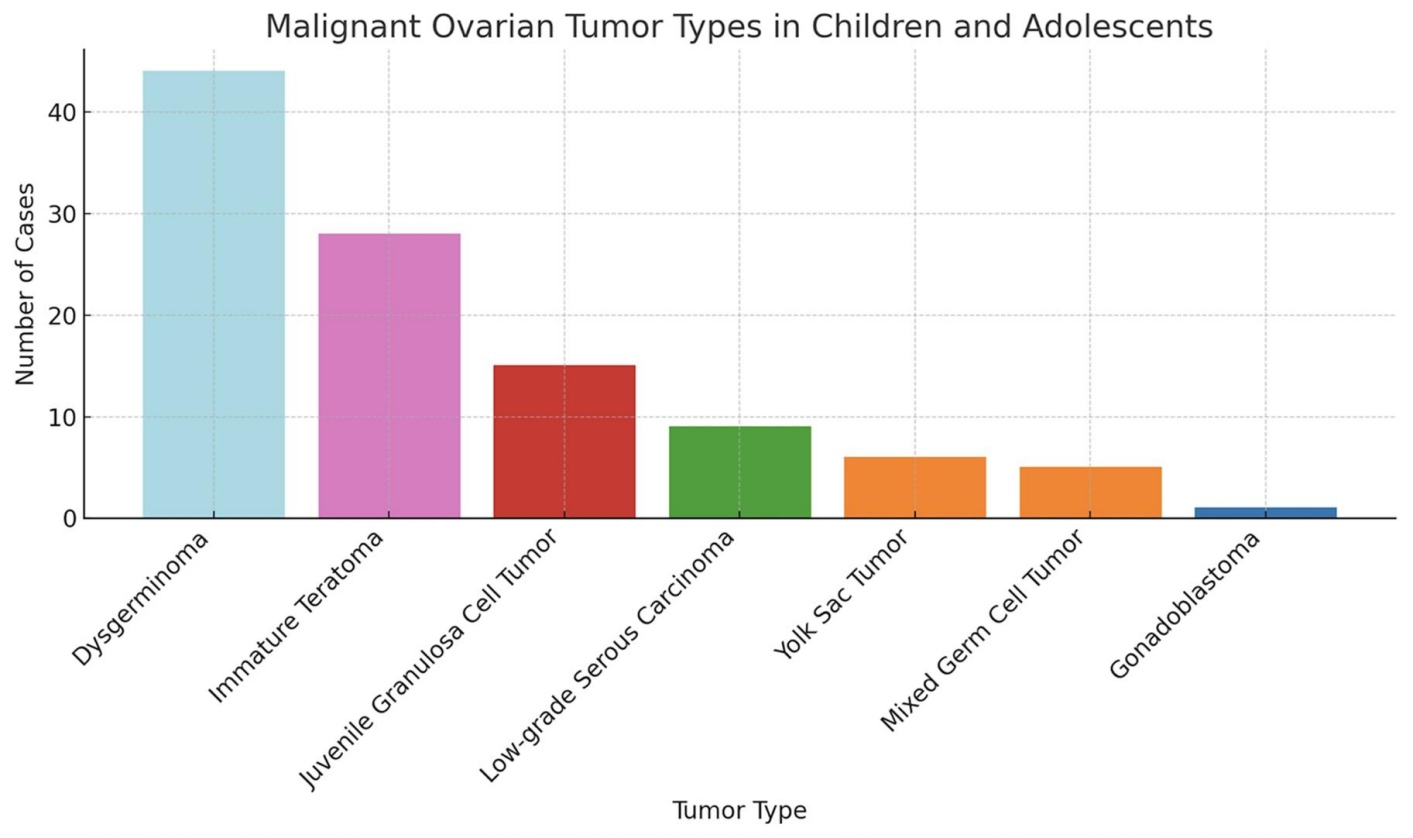
Distribution of patients diagnosed with malignancy.

The histopathological distribution of diagnoses is provided in Table 3. Among malignant lesions, the most common were dysgerminoma, immature teratoma, and juvenile granulosa cell tumor (Figure 1). The distribution of malignancy types among patients is illustrated in Figure 2.

**Table 3:** Distribution of pathological diagnoses.

Pathological diagnosis	Number of patients	Percentage (%)
Non-Neoplastic		
Follicular cyst	548	48.5
Ovarian torsion (without tumor)	114	10.11
Corpus luteum cyst	112	9.93
Endometrioma	98	8.69
Neoplastic—Benign		
Mature cystic teratoma	106	9.4
Serous cystadenoma	13	1.15
Mucinous cystadenoma	11	0.98
Fibrothecoma	10	0.89
Borderline serous tumor	5	0.44
Borderline mucinous tumor	3	0.27
Neoplastic—Malignant		
Dysgerminoma	44	3.9
Immature teratoma	28	2.48
Juvenile granulosa cell tumor	15	1.33
Low-grade serous carcinoma	9	0.8
Yolk sac tumor	6	0.53
Mixed germ cell tumor	5	0.44
Gonadoblastoma	1	0.09

Regarding tumor markers, AFP demonstrated a specificity of 88%, and β-hCG showed a specificity of 90% (Table 4).

**Table 4:** Tumor marker performance in predicting malignancy.

Tumor marker	Sensitivity (%)	Specificity (%)	PPV (%)	NPV (%)	*p*-value
AFP	92	88	86	93	0.001
*β*-hCG	85	90	83	89	0.005
LDH	78	82	76	83	0.02
CA-125	60	75	58	77	0.08
Inhibin B	70	85	72	84	0.03

## Discussion

Ovarian masses in childhood and adolescence represent a significant clinical challenge due to their broad histopathological diversity and the necessity of preserving reproductive function in this age group. This retrospective analysis evaluates the diagnostic and surgical approaches implemented at our center, along with the clinical outcomes observed in this patient population. Our findings align with current literature, indicating that the majority of ovarian masses in children and adolescents are benign; however, there is a noteworthy risk of malignancy, particularly among germ cell tumors (19–23). As widely reported, mature cystic teratoma is the most commonly encountered benign lesion (21). Histological distribution can also vary by age, with dermoid and simple cysts being more prevalent in adolescents and endometriomas more commonly reported in young adults (22).

Although patient history and physical examination are fundamental to the diagnostic process, imaging techniques and tumor markers play a critical role in increasing diagnostic accuracy. Intermittent abdominal pain has been suggested as a predictive symptom of ovarian torsion, and ultrasonography remains the first-line imaging modality (6). In cases of suspected malignancy, advanced imaging methods such as computed tomography (CT) and magnetic resonance imaging (MRI) offer greater diagnostic value. MRI, in particular, has been shown to provide superior sensitivity and specificity in differentiating between benign and malignant lesions (9). CT is highly effective in diagnosing mature teratomas due to its ability to detect characteristic features such as fat and calcification.

Tumor markers are also useful in predicting malignancy. Alpha-fetoprotein (AFP) is especially specific for malignant germ cell tumors such as yolk sac tumors (9). In our study, AFP demonstrated a specificity of 88%, while β-hCG showed 90%, supporting the utility of serum marker panels in differential diagnosis.

The cornerstone of surgical management is to preserve gonadal tissue as much as possible while adhering to oncological principles (13). Ovarian-sparing procedures such as cystectomy are recommended for benign lesions. Laparoscopic surgery has been shown to better preserve ovarian tissue compared to open procedures (12), with literature reporting significantly higher ovarian preservation rates with laparoscopy than laparotomy (55.66% vs. 17.44%) (12). In cases of suspected malignancy, laparotomy and, if necessary, oophorectomy are often performed. Early diagnosis is vital in torsion cases; even in the presence of ischemic appearance, detorsion can be effective in preserving ovarian viability. Takayasu et al. reported that elevated leukocyte counts may be a negative predictor of ovarian salvageability (6).

Our findings emphasize the importance of individualized surgical planning based on age, tumor size, and malignancy risk. Laparoscopic surgery, performed in 20% of our cohort, was associated with higher ovarian preservation rates and reduced recovery times, consistent with existing literature (12, 13). Nevertheless, open surgery remains essential in cases with large, suspicious masses or in emergency situations such as ovarian torsion. The decision between ovarian-sparing procedures and oophorectomy must carefully balance oncologic safety and fertility preservation (11).

Unfortunately, our retrospective data did not allow for a standardized follow-up protocol across all patients. Therefore, recurrence rates and long-term fertility outcomes could not be thoroughly assessed. This represents a limitation of the study and underscores the need for prospective multicenter studies with structured long-term follow-up to evaluate outcomes such as recurrence, hormonal function, and reproductive potential (15, 19).

Long-term follow-up is crucial in pediatric and young adult patients with ovarian tumors, particularly to assess recurrence risk and future fertility potential. In our cohort, recurrence was observed in 31% of patients with borderline ovarian tumors (BOT) following fertility-sparing surgery (FSS). Among patients with immature teratomas, the recurrence rate was 33% in grade 2–3 cases and only 5% in grade 1 tumors. Additionally, 12% of patients with malignant germ cell tumors experienced recurrence. These findings are consistent with previous studies, which report recurrence rates of 29%–33% in BOTs and variable recurrence in immature teratomas depending on grade (9, 13). However, the retrospective nature of our study and limitations in long-term follow-up data constrain the generalizability of these results. Future prospective, multicenter studies with standardized follow-up protocols are needed to more accurately define recurrence patterns and long-term reproductive outcomes in this population.

Additionally, pediatric ovarian tumors may be associated with specific genetic syndromes. The risk of secondary neoplasms following initial malignancy is gaining increasing attention in relation to genetic predispositions (19). In our cohort, one patient diagnosed with juvenile granulosa cell tumor also had Ollier syndrome, supporting this consideration. Therefore, genetic evaluation should be an integral part of oncologic management (17).

Future research should aim to develop evidence-based standardized algorithms for the management of pediatric and adolescent ovarian masses through multicenter, prospective studies. It is also essential to assess the long-term fertility outcomes associated with various surgical techniques, especially laparoscopy, and to explore the role of genetic predisposition in greater depth. Tailored follow-up strategies should be developed for patients identified as high-risk.

## Conclusıon

Ovarian tumors in the pediatric and adolescent population represent a heterogeneous group of histopathological entities. While rare, these tumors carry a risk of malignancy. This study provides an in-depth analysis of a large cohort of children and young adolescents diagnosed and treated for ovarian tumors at our institution.

## Lımıtatıon

This study has several limitations. Its retrospective nature and single-center data restrict the generalizability of the findings. In certain cases, limited access to advanced imaging (e.g., MRI) or laparoscopy may not fully reflect the true utility of these diagnostic and therapeutic modalities. Moreover, surgical decision-making processes, particularly regarding ovarian preservation, may have been influenced by subjective factors. Additionally, the lack of data on fertility outcomes among adult patients represents a limitation and should be considered when interpreting the results.

## Data Availability

The original contributions presented in the study are included in the article/Supplementary Material, further inquiries can be directed to the corresponding author.
